# Inverse Levels of Adiponectin in Type 1 and Type 2 Diabetes Are in Accordance with the State of Albuminuria

**DOI:** 10.1155/2015/372796

**Published:** 2015-05-18

**Authors:** Spomenka Ljubic, Anamarija Jazbec, Martina Tomic, Ante Piljac, Dubravka Jurisic Erzen, Branko Novak, Snjezana Kastelan, Marijana Vucic Lovrencic, Neva Brkljacic

**Affiliations:** ^1^Department of Endocrinology and Metabolic Disease, Vuk Vrhovac University Clinic, Merkur University Hospital, Zajceva 19, 10000 Zagreb, Croatia; ^2^Faculty of Forestry, University of Zagreb, Svetosimunska 25, 10000 Zagreb, Croatia; ^3^Department of Ophthalmology, Vuk Vrhovac University Clinic, Merkur University Hospital, Zajceva 19, 10000 Zagreb, Croatia; ^4^Department of Internal Medicine, Rijeka University Hospital Center, Kresimirova 42, 51000 Rijeka, Croatia; ^5^Department of Diabetes, Vuk Vrhovac University Clinic, Merkur University Hospital, Zajceva 19, 10000 Zagreb, Croatia; ^6^Department of Ophthalmology, Dubrava Clinical Hospital, Avenija Gojka Suska 6, 10000 Zagreb, Croatia; ^7^Department of Laboratory Medicine, Merkur University Hospital, 10000 Zagreb, Croatia; ^8^Department of Cardiology, Merkur University Hospital, Zajceva 19, 10000 Zagreb, Croatia

## Abstract

*Aims.* To investigate the behaviour of adiponectin (ApN) in patients with type 1 and type 2 diabetic nephropathy.* Methods.* ApN and inflammatory and other markers of the metabolic syndrome were compared across diabetes types, albumin excretion rate (AER), and creatinine clearance (CrCl) categories in 219 type 1 and type 2 diabetic patients.* Results.* Significant differences among ApN levels according to AER were found in both types of diabetes (*F* = 8.45, df = 2, *P* < 0.001). With the progression of albuminuria, ApN increased in type 1 and decreased in type 2 diabetes. Patients with decreased CrCl had higher ApN levels than those with normal CrCl in either type of diabetes (*F* = 12.7, df = 1, *P* < 0.001). The best model for ApN (*R* ^2^ = 0.9002) obtained from stepwise regression in type 1 diabetes included CrCl, BMI, WBC, CRP, and age, while in type 2 diabetes (*R* ^2^ = 0.2882) it included ppPG, LDL, and UA.* Conclusion.* ApN behaved differently in relation to albuminuria, increasing with its progression in type 1 diabetes and decreasing in type 2 diabetes. It was however increased in the subgroups with decreased CrCl in both types of diabetes. Albuminuria seems to be more important than renal insufficiency in the definition of ApN levels in type 1 and type 2 diabetes.

## 1. Introduction

Adiponectin (ApN) has an impact on endothelial cell function by its anti-inflammatory properties and stimulation of nitric oxide production [1, 2]. On the other hand, dyslipidemia is characterized by increased serum triglycerides and decreased high-density lipoprotein cholesterol (HDL-C), which correlates with low ApN levels [3]. Dyslipidemia as part of the metabolic syndrome is a risk factor for endothelial dysfunction and atherosclerosis as well. Its levels are significantly higher in women than in men, probably due to variations in sex hormones during lifetime [3, 4]. Although serum ApN in healthy people might be associated with vascular function independently of insulin resistance, increased inflammatory markers are connected with an increase in insulin resistance in patients with impaired glucose tolerance, type 2 diabetes, and obesity, who also have low circulating ApN concentrations [2, 5–7]. Nevertheless, due to its insulin sensitizing action, ApN seems to have a role in both insulin resistance and vascular protection [8]. Furthermore, because low ApN affects dyslipidemia, inflammation, insulin sensitivity, and vascular protection, it is important in the onset of cardiovascular events [9].

As an anti-inflammatory mediator, ApN might also be responsible for the prevention of diabetic microangiopathy. In type 1 diabetic patients nephropathy correlates with increased ApN levels [3, 10]. Recent prospective studies have found a link between hyperadiponectinemia and mortality in chronic kidney disease. ApN has been reported to play a protective role in male wild-type mice by reducing albuminuria through an effect on podocytes through the AMP-activated protein kinase (AMPK) pathway [7, 11].

A family history of diabetes could be associated with hypoadiponectinemia. Adiponectin gene polymorphisms have been determined to be associated with a risk of diabetic nephropathy [12]. Our previous study has demonstrated significantly higher adiponectin levels in type 1 diabetes as compared with type 2 diabetes and also identified C-peptide as a significant determinant of this difference [13].

The aim of the present study was to investigate the relationship of adiponectin and other markers of the metabolic syndrome with nephropathy in patients with type 1 and type 2 diabetes.

## 2. Materials and Methods

The study protocol was approved by the hospital's ethics committee. The patients received both written and oral information about the study and signed a written informed consent.

### 2.1. Patients

A total of 219 patients treated at our outpatient department were included in the study: 87 with type 1 diabetes and 132 with type 2 diabetes. Blood samples were taken after 12 hr fast. Patients with type 2 diabetes were on oral hypoglycemic agents and/or diet, and patients with type 1 diabetes were on either intensive insulin treatment or 2 to 3 doses of premixed insulin. Diabetes mellitus was defined according to the American Diabetes Association classification [14]. Patients with malignancies and immunologic and infectious inflammatory diseases, pregnant women, and patients receiving corticosteroids or cytostatics were not included in the study. In all patients, fundoscopy was performed to determine the presence of retinopathy and to decide on further diagnostic procedures in patients with albuminuria and without retinopathy.

Clinical and laboratory markers of diabetes, obesity, and metabolic syndrome included age, diabetes duration, body mass index (BMI), ApN, C-reactive protein (CRP), fibrinogen (FIB), homocysteine (HCY), creatinine clearance (CrCl), creatinine, systolic blood pressure (SBP), diastolic blood pressure (DBP), fasting (fPG) and postprandial plasma glucose (ppPG), glycated hemoglobin (A1c), liver function tests (aspartate aminotransferase [AST], alanine aminotransferase [ALT], and gamma-glutamyl transpeptidase [GGT]), lipids (high density lipoprotein [HDL-C], low density lipoprotein [LDL-C], and triglycerides [TG]), ferritin, uric acid (UA), creatine phosphokinase (CPK), and leucocyte count (WBC) were determined. Patients were assigned to subgroups based on albumin excretion rate (AER) (<30 [normoalbuminuria], 30–300 [microalbuminuria], >300 mg/24 h [macroalbuminuria]), and CrCl (normal >0.83 mL/sec for women and >1.17 mL/sec for men). AER and CrCl were calculated from three consecutive urine sample collections. Blood pressure was measured after five minutes of supine rest and mean values of three measurements were used in statistical analysis. Previous myocardial infraction and stroke were also determined.

### 2.2. Laboratory Tests

Serum ApN was measured by sandwich ELISA (DRG, Marburg, Germany), plasma FIB by the Clauss method, and hs-CRP by an immunoturbidimetric assay on an Olympus AU600 analyzer (Beckman-Coulter, Brea, CA, USA). Hemoglobin A1c was measured by an automated immunoturbidimetric procedure on a dedicated analyzer (Integra, TinaQuant, Roche Diagnostics, Hoffmann-LaRoche, Basel, Switzerland) with results traceable to the NGSP-standard.

HCY in EDTA plasma was measured by an automated chemiluminescence assay (Advia Centaur, Siemens Diagnostic Solutions, Tarrytown, NY, USA). Cholesterol, TG, UA, and glucose were analyzed using standard enzymatic procedures and HDL-C using a homogeneous assay on an automated analyzer (Olympus AU600, Beckman-Coulter, Brea, CA, USA).

### 2.3. Data Analysis

All variables, age, diabetes duration, BMI, ApN, CRP, FIB, HCY, CrCl, creatinine, SBP, DBP, fPG, ppPG, A1c, AST, ALT, GGT, HDL-C, LDL-C, and TG, ferritin, UA, CPK, and WBC, were analyzed using descriptive statistics. Type error *I*(*α*) of 0.05 was considered statistically significant.

Differences between type 1 and type 2 diabetes were tested using Student's *t*-test or Mann-Whitney* U* test if assumption of homogeneity of variance was not satisfied. Difference in ApN between the groups according to AER (<30, 30–300, >300) and CrCl (normal >0.83 mL/sec for women and >1.17 mL/sec for men) and their interactions were tested using analysis of variance (ANOVA). If a significant difference was observed, Tukey's HSD post hoc test was used to determine which groups were significantly different from each other. Stepwise regression was used to detect main predictors of ApN in DM groups. Student's *t*-test, Mann-Whitney* U* test, and ANOVA were performed using STATISTICA 8 and stepwise regression using SAS 9.1 [15]. The graphs were created using STATISTICA [16].

## 3. Results

ApN and HDL were significantly increased in type 1 diabetes in comparison with type 2 diabetes, whereas CRP, FIB, HCY, and GGT were significantly increased in DM2 (Table 1).

**Figure 1:**
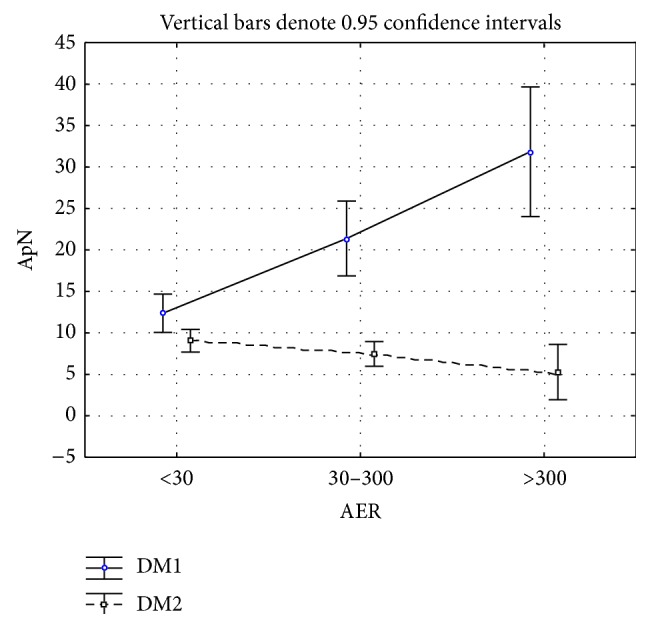
ApN mean values and 95% confidence intervals in patients with type 1 and type 2 diabetes according to AER.

Statistically significant differences among ApN levels according to AER were found (*F* = 8.45, df = 2, *P* < 0.001) between type 1 diabetes (<30 = 12.37 ± 6.62, 30–300 = 21.38 ± 7.98, and >300 = 31.85 ± 18.05) and type 2 diabetes (<30 = 9.05 ± 5.63, 30–300 = 7.46 ± 4.58, and >300 = 5.26 ± 3.3) (Figure 1). The difference in duration of disease between type 1 and type 2 diabetic patients was not observed (6.02 ± 5.25 yrs and 7.14 ± 7.51 yrs) (*P* = 0.97). There were also significant differences in ApN between the types of diabetes (*F* = 73.402, df = 1, *P* < 0.001) and in the interaction between the type of diabetes and AER (*F* = 18.12, df = 2, *P* < 0.001), indicating that ApN did not behave comparably across AER categories in both types of diabetes. In type 1 diabetes ApN levels increased with an increase in AER, whereas in type 2 they were found to decrease (Table 2, Figure 1). Patients with type 1 diabetes had significantly higher ApN values than those with type 2 diabetes. Tukey's post hoc test pointed to a significant difference in ApN between type 1 diabetic subgroups with normoalbuminuria and microalbuminuria and those with normoalbuminuria and macroalbuminuria, as well as between type 1 and type 2 subgroups with normoalbuminuria and macroalbuminuria (Table 3). Patients with type 1 and type 2 diabetes differ (Student's *t*-test) in the occurrence of myocardial infarction (type 1: 3.4% and type 2: 4.5%) and stroke (type 1: 2.3% and type 2: 5.3%).

**Figure 2:**
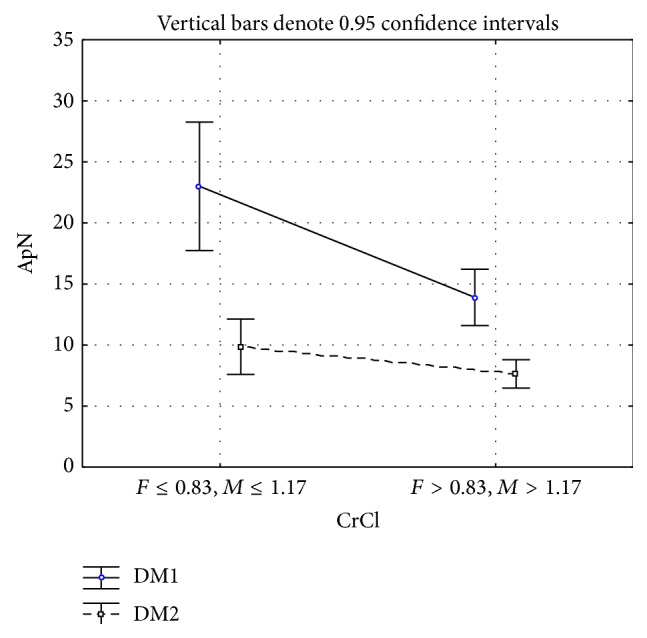
ApN mean values and 95% confidence intervals in patients with type 1 and type 2 diabetes according to CrCl.

In a model for ApN as a dependent variable and the type of diabetes, CrCl, and the interaction between the type of DM and CrCl as factors, a statistically significant difference was found for all analyzed factors (Figure 2). ApN levels according to CrCl (*F* = 12.7, df = 1, *P* < 0.001) were higher in patients with decreased CrCl (1) than in those with normal CrCl (2). In type 1 diabetic patients with normal CrCL (2) ApN levels were 13.9 ± 7.93, and in patients with decreased CrCL (1) they were 23 ± 12.8. In type 2 diabetic patients with normal CrCl (2), those levels were 7.63 ± 4.76 and in patients with decreased CrCl (1) they were 9.86 ± 6.25 (Table 2). Although ApN was not significantly increased in the group with decreased CrCl, post hoc results pointed to a significant increase in ApN in type 1 diabetes subgroups with normal and decreased CrCL as compared with type 2 diabetes (Table 3).

The best model for ApN (*R* ^2^ = 0.9002) obtained from stepwise regression in type 1 diabetes included CrCl, BMI, WBC, CRP, and age, while in type 2 diabetes the best model (*R* ^2^ = 0.2882) included ppPG, LDL, and UA (Table 4).

In type 1 diabetes ApN correlated significantly (*P* < 0.05) with HCY (*r* = 0.57), CrCL (*r* = −0.61), AER (*r* = 0.61), and creatinine (*r* = 0.40), and in type 2 diabetes it correlated with HCY (*r* = 0.25), CrCl (*r* = −0.22), creatinine (*r* = 0.20), and diastolic BP (*r* = −0.19).

## 4. Discussion

ApN and inflammatory factors are inversely correlated [6], which was confirmed by the observed significant differences in ApN, CRP, FIB, and HCY according to the type of diabetes. Levels of ApN and HDL were significantly increased in type 1 diabetes in comparison with type 2 diabetes, whereas CRP, FIB, HCY, and GGT were significantly increased in type 2 diabetes, indicating that this type of diabetes is an inflammatory state. This is in agreement with the reports on decreased ApN levels in patients with prediabetes and type 2 diabetes [17] and a negative association between plasma ApN and the levels of CRP and WBC [18].

GGT, a marker of the metabolic syndrome and fatty liver, was significantly increased in type 2 diabetes in comparison with type 1 diabetes. A significant association between GGT and insulin resistance has previously been demonstrated in diabetic patients, introducing GGT as one of the markers for metabolic syndrome [19]. Serum ApN and tumor necrosis factor-*α* are independent predictors of liver steatosis, and ApN has additionally been found to be an independent predictor of response to chronic hepatitis C therapy [20].

Recent reports have pointed to an interrelation between C-peptide levels and ApN production in adipocytes [13]. In nonobese subjects ApN has been shown to correlate better with *β*-cell function than with insulin sensitivity [21]. The relationship between intensive insulin treatment and ApN is still not clear. The DIGAMI study has demonstrated that diabetic patients with acute myocardial infarction on intensive insulin treatment have a better prognosis and an absolute reduction in mortality [22]. The effect of intensive insulin treatment on ApN levels has been discussed as a possible explanation [23]. Several authors have observed that intensive insulin treatment normalized elevated serum C-peptide and increased circulating ApN levels, thus improving insulin sensitivity [24, 25]. This is in agreement with our results which showed that diabetic patients on intensive insulin treatment had increased ApN and decreased CRP levels.

In this study, diastolic BP correlated significantly with ApN in type 2 diabetes. Low ApN level could be considered to be a marker that predicts arterial hypertension and stiffness as a result of impaired vasodilation caused by decreased nitric oxide (NO) production in insulin resistant patients [26]. A correlation between epicardial adipose tissue expression, low ApN level, and hypertension has already been reported [2]. Although HCY is also a risk factor for cardiovascular diseases, its association with nephropathy seems even more important. The present study showed a correlation between HCY and ApN levels in both types of diabetes and an increased HCY level in type 2 diabetes. Seshardi and coworkers have shown that HCY and CRP are associated with the onset and progression of albuminuria as measured by the albumin/creatinine ratio and with the promotion of inflammatory state but through different mediators of inflammation [27].

Our results did not reveal a correlation between ApN and BMI either in type 1 diabetic patients or in type 2 patients, which corresponds to the lack of such a correlation reported in Japanese men [10, 28]. A study of the association of ApN with insulin resistance and dyslipidemia has demonstrated that ApN does not correlate with overall obesity but with subcutaneous abdominal fat. Furthermore, the impact of overall obesity on ApN production has also been found to be less significant than that of epicardial fat tissue [2].

As reported in the literature, ApN is associated with endothelial protection [8]. Its presumed protective role, however, is not in agreement with the finding of increased ApN levels in type 1 diabetic patients and especially in those with advanced stages of nephropathy. Higher ApN levels in type 1 diabetes have been reported to be associated with the onset and progression of microalbuminuria [10]. A possible explanation could be that ApN can change the integrity of endothelial junctions and induce NO production, thus affecting hyperfiltration [29]. Increased ApN levels in patients with renal failure and proteinuria could be attributed to increased ApN production and reduced clearance in renal failure [30]. Increased ApN has been hypothesized to be a marker of cachexia and catabolism in subjects with renal failure, type 1 diabetes, or weight loss [31]. Another reason why ApN is increased in nephropathy could be that, belonging to the soluble collagen family, it accumulates in the subintimal space of the arterial wall through its interaction with collagens in the vascular intima and consequently attenuates TNF-*α*–induced expression of adhesion molecules in endothelial cells [2]. Studies in male wild-type mice have demonstrated the protective role of ApN in affecting podocytes through the AMPK pathway, thus reducing albuminuria [7, 11].

In this study, statistically significant differences in ApN levels among different stages of albuminuria were found between type 1 diabetes and type 2 diabetes (Figure 1). There were also significant differences in ApN between the types of diabetes and in the interaction between the type of diabetes and AER. Patients with type 1 diabetes had significantly higher ApN than those with type 2 diabetes at any AER level. Significant differences in ApN were also found between type 1 diabetic subgroups with normoalbuminuria and microalbuminuria and with normoalbuminuria and macroalbuminuria. Such differences were also observed in the subgroups with normoalbuminuria and macroalbuminuria between type 1 and type 2 diabetic patients (Table 3). ApN increases with the progression of albuminuria in type 1 diabetic patients, which is concordant with the observed hyperadiponectinemia in patients with chronic kidney disease [32]. High plasma ApN concentrations decrease after renal transplantation, suggesting that renal insufficiency may either have an effect on ApN clearance and/or stimulate ApN production. The metabolic state improves after transplantation [33]. Our patients with type 2 diabetes showed a decrease in ApN levels with the progression of albuminuria and a rise in other inflammatory markers, which pointed to an increase in inflammation accompanying albuminuria. The decrease in ApN in type 2 diabetes could also be explained by obesity, as suggested by Guebre-Egziabher and coworkers [34].

In a study of 50 patients with type 2 diabetes, ApN was increased in subjects with macroalbuminuria and inversely correlated with creatinine levels. The authors have concluded that macroalbuminuria is superior to impaired renal function in determining the level of ApN [35]. The decrease in ApN relative to the progression of albuminuria in patients with type 2 diabetes observed in our study confirmed results of previous studies which have demonstrated that low circulating ApN that accompanies proteinuria regardless of the degree of renal function impairment is an important predictor of endothelial dysfunction [36].

ApN levels were higher in patients with decreased CrCl than in those with increased CrCl in both types of diabetes (Table 2). A model for ApN as a dependent variable and the type of diabetes mellitus, CrCl, and the interaction between the type of diabetes and CrCl as factors revealed statistically significant differences for all analyzed factors (Figure 2). ApN was significantly higher in type 1 diabetes than in type 2 diabetes in both normal and decreased CrCl subgroups (Table 3). This increase could also be associated with a possible role of C-peptide as one of the determinants responsible for the difference in ApN levels between type 1 and type 2 diabetes [13, 21–24]. A high plasma ApN level observed in a mice renal failure model has been explained by a low clearance rate [7]. The decrease in CrCl in our study population could also be responsible for changes in ApN levels. CrCl-related ApN in the present study behaved the same in both types of diabetes, in contrast to the behavior it exhibited in relation to albuminuria. This points to the importance of albuminuria in comparison with CrCl in the determination of ApN level, as concluded by Ran and coworkers [35].

In patients with type 1 diabetes ApN was best predicted by CrCl, BMI, WBC, CRP, and age. In patients with type 2 diabetes, the main predictors were ppPG, LDL-cholesterol, and UA (Table 4). Besides other known predictors such as CRP, LDL-cholesterol, and UA, WBC could be held responsible for the development of atherosclerosis [18]. Higher UA levels have been proven to be a protective antioxidant mechanism in hyperglycemic states and obesity. They have also been connected to microvascular complications in diabetes [37]. There is evidence that plasma ApN level is associated with hyperlipidemia and a consequently increased cardiovascular risk in patients with renal insufficiency [38]. Although there was a difference in occurrence of myocardial infarction and stroke between groups of patients with type 1 and type 2 diabetes, the number of patients limited the comparison between groups according to albuminuria levels. As it is known, albuminuria and ApN are among markers of cardiovascular disease. In this study, ApN behaved differently in different type of diabetes which could be among the reasons for different cardiovascular risk [39].

Our finding of decreased HDL-C and ApN in type 2 diabetes in comparison with type 1 diabetes corresponds to the reports hypothesizing that low ApN might be a trigger for dyslipidemia [25], the finding that LDL was among the main predictors of ApN in type 2 diabetes further corroborating the hypothesis.

## 5. Conclusion

ApN was increased in type 1 and type 2 diabetic subgroups with decreased CrCl but behaved differently in relation to albuminuria, showing an increase with the progression of albuminuria in type 1 diabetes and a decrease in type 2 diabetes. It was higher in the subgroups with normoalbuminuria and macroalbuminuria in type 1 diabetes than in comparable type 2 subgroups. It seems that the interaction between various degrees of renal insufficiency and albumin loss could be important on ApN levels. Increased levels of ApN in type 1 diabetes could be explained by a positive feedback loop, where loss of ApN due to albuminuria causes increased synthesis of ApN. This in turn suggests a protective anti-inflammatory action of ApN. Other inflammatory markers investigated in this study were found to be decreased in type 1 diabetes. The interaction between inflammation as part of the metabolic syndrome and ApN level is important in the development of nephropathy, possibly being responsible for different courses of nephropathy in the two types of diabetes. We can expect that patients with different types of diabetes might be under different risk for cardiovascular disease, in part because of a period of undiagnosed type 2 diabetes but mostly because of the difference in inflammation, adiponectin level, and presentation of metabolic syndrome in each type of diabetes. ApN behavior opens many questions which will be answered in futures studies.

## Figures and Tables

**Table 1:** Differences between biochemical data of the study groups according to type of diabetes.

Variable	Group 1: DM1 *t*-test			Group 2: DM2 *t*-test			*t*-value	df	*P*
	Mean 1	Std. Dev.	*N* 1	Mean 2	Std. Dev.	*N* 2			
Hs-CRP (mg/L)	2.06	3.45	87	3.66	4.17	131	−1.98	160	0.049
FIB (g/L)	3.76	1.43	87	4.73	1.22	132	−3.86	161	<0.001
Ferritin (*μ*g/mL)	135.2	136.9	84	155.9	122.75	89	−0.72	111	0.474

	Mann-Whitney *U* test			Mann-Whitney *U* test			*U*	*Z*	*P*
	Mean 1	Std. Dev.	*N* 1	Mean 2	Std. Dev.	*N* 2			

ApN (*μ*g/mL)	15.37	9.27	87	8.07	5.15	131	886.5	4.87	<0.001
HCY (*μ*mol/mL)	11.1	2.92	87	15.6	6.92	132	1105.5	−3.98	<0.001
Lp(a) (mg/dL)	23.52	21.65	87	39.9	52.31	119	1172	−1.38	0.167
HDL-C (mmol/L)	1.6	0.5	87	1.34	0.32	132	1314	3.09	0.002
AST (U/L)	25.67	10.9	85	22.86	7.09	131	1641	1.40	0.160
ALT (U/L)	31.12	17.5	87	29.05	13.4	131	1956	0.32	0.751
GGT (U/L)	23.03	13.6	85	37.37	30.1	131	1171	−3.45	0.001
UA (*μ*mol/L)	283.13	83.8	85	383.3	350.43	132	1127	−3.68	<0.001

DM1, type 1 diabetes; DM2, type 2 diabetes.

**Table 2:** Results of ANOVA for ApN as dependent variables according to AER, type of diabetes, CrCl, and their interactions.

Dependent variable	Factors	Sums of squares	Degree of freedom	Mean squares	*F*	*P*
ApN	Type of DM	2299.98	1	2299.98	73.4	<0.0001
	AER	529.55	2	264.78	8.45	0.0003
	DM ∗ AER	1135.66	2	567.83	18.12	<0.0001

ApN	Type DM	1321.04	1	1321.04	37.32	<0.0001
	CrCl	449.71	1	449.71	12.7	0.0005
	DM ∗ CrCl	165.76	1	165.76	4.68	0.032

**(a):** 

Cell number	Type of DM	AER mg/24 h	{1}	{2}	{3}	{4}	{5}
{1}	1	<30					
{2}	1	30–300	0.0060				
{3}	1	>300	0.0001	0.1972			
{4}	2	<30	0.1422	<0.0001	<0.0001		
{5}	2	30–300	0.0056	<0.0001	<0.0001	0.6316	
{6}	2	>300	0.0071	<0.0001	<0.0001	0.2991	0.8415

**(b):** 

Cell number	Type of DM	CrCl mL/s	{1}	{2}	{3}
{1}	1	*F* ≤ 0.83, *M* ≤ 1.17			
{2}	1	*F* > 0.83, *M* > 1.17	0.0094		
{3}	2	*F* ≤ 0.83, *M* ≤ 1.17	<0.0001	0.0642	
{4}	2	*F* > 0.83, *M* > 1.17	<0.0001	<0.0001	0.3083

**Table 4:** Results of stepwise regression analysis for ApN as a dependent variable in DM1 and DM2.

	Variable	Parameter estimate	Standard error	*F*	*P*	Partial *R* ^2^	*R* ^2^
DM1	Age	0.265	0.064	17.08	0.0020	0.0608	0.9063
	BMI	−2.073	0.294	49.86	<0.0001	0.3454	
	Hs-CRP	0.727	0.251	8.41	0.0158	0.0441	
	CrCl	−10.975	2.035	29.06	0.0003	0.3197	
	WBC	1.830	0.503	13.24	0.0046	0.0593	

DM2	ppPG	−0.603	0.284	4.53	0.0426	0.1418	0.2882
	LDL	2.035	1.039	3.83	0.0606	0.0862	
	UA	0.018	0.012	2.28	0.1423	0.0602	

All variables left in the model are significant at the 0.15 level.
